# The Use of NPWT-i Technology in Complex Surgical Wounds

**DOI:** 10.7759/cureus.920

**Published:** 2016-12-08

**Authors:** Paula Rupert, Robert A Ochoa, Laurie Punch, Jeffrey Van Epps, Sherilyn Gordon-Burroughs, Sylvia Martinez

**Affiliations:** 1 Surgery, Houston Methodist Hospital

**Keywords:** complex wounds, wound care, surgical wounds, negative pressure wound therapy with instillation

## Abstract

Advanced wound management of complex surgical wounds remains a significant challenge as more patients are being hospitalized with infected wounds. Reducing recurrent infections and promoting granulation tissue formation is essential to overall wound healing. Wounds with acute infection and critical colonization require advanced multimodal approaches including systemic antibiotics, surgical debridement, and primary wound care. The goal in surgical wound management is to optimize clinical outcomes such as time to wound closure and functional recovery. A review of current literature suggests that negative pressure wound therapy with instillation (NPWT-i) is a viable adjunct therapy in the management of infected wounds especially in patients with medical comorbidities. The aim of this case series is to highlight the ability of NPWT-i as adjunct to prepare the wound bed for closure on infected surgical wounds that would normally require multiple operations to obtain source control.

## Introduction

Advanced wound management of complex surgical wounds remains a significant challenge as more patients are being hospitalized with infected wounds. Reducing recurrent infections and promoting granulation tissue formation is essential to overall wound healing [1]. Wounds with acute infection and critical colonization require advanced multimodal approaches including systemic antibiotics, surgical debridement, and primary wound care. The goal in surgical wound management is to optimize clinical outcomes such as time to wound closure and functional recovery. A review of current literature suggests that negative pressure wound therapy with instillation (NPWT-i) is a viable adjunct therapy in the management of infected wounds especially in patients with medical comorbidities.

A retrospective cohort study by Gabriel, et al. [2] compared the use of NPWT-i with silver nitrate in 15 patients with complex infected wounds compared to a retrospective control population (n = 15) who were managed with moist gauze dressings. The inclusion criteria for both groups included trunk and extremity wounds with documented qualitative cultures with greater than 105 organisms, age greater than 40 years and documented presence of necrotic tissue. Results: the NPWT-i group had fewer treatment days (9·9 ± 4·3 days versus 36·5 ± 13·1 days, P < 0·001), quicker wound closure (13·2 ± 6·8 versus 29·6 ± 6·5 days, P < 0·001), and decreased inpatient lengths of stay (14·7 ± 9·2 versus 39·2 ± 12·1 days, P < 0·001).

Timmers, et al. [3] performed a retrospective cohort study utilizing NPWT-i in patients (n = 30) with post-traumatic osteomyelitis. The matched control group (n = 94) received standard wound care, which included the use of antibiotic beads. The instillation solution utilized was a 0·04% polyhexanide solution for 10–15 minutes dwell time, with negative pressure of 300–600 mmHg applied through a prototype negative pressure therapy system. Results: the infection recurrence rate was 10% for the NPWT-i group compared with 58·5% in the control group (P < 0·0001).

In a study by Brinkert, et al., [4] a prospective clinical study of 131 patients with wounds were treated with NPWT-i using saline. Patients aged 18 years or older with an infected wound or wound at risk of infection were eligible to receive NPWTi. Patients who were already being treated with conventional NPWT were also eligible to receive NPWT-i. Results: in 98% of the cases, the wounds could be closed following debridement with the use of NPWT-i adjunctive therapy. The mean duration of NPWT-i was 12·19 days.

Goss, et al. [5] investigated 13 patients with 16 chronic lower leg and foot wounds treated with debridement. The patients were randomized to receive either one week of NPWT-i with bleach solution or one week of regular negative pressure wound therapy (NPWT). Quantitative cultures were taken preoperatively after sterile preparation and draping of the wound site (POD # 0, pre-op), postoperatively once debridement was completed (POD # 0, post-op), and on POD 7 after operative debridement. Results: after operative debridement (postoperative day 0) there was a mean of three (±1) types of bacteria per wound. The mean absolute reduction in bacteria for the NPWT-i group was 10.6 × 10(6) bacteria per gram of tissue while there was a mean absolute increase in bacteria for the NPWT group of 28.7 × 10(6) bacteria per gram of tissue—a statistically significant reduction in the absolute bioburden in those wounds treated with NPWT-i (p = 0.016) was found.

Kim et al. [6] performed a retrospective cohort study with 142 patients hospitalized for acutely infected wounds comparing NPWT (n = 74) and NPWT-i (n = 74). Included patients presented similarly with multiple comorbidities such as diabetes mellitus, peripheral vascular disease, and end-stage renal disease. All received systemic antibiotics and surgical debridement. Data from patients treated with two different dwell times and negative pressure durations (n = 34, six-minute dwell time and 3·5 hours of negative pressure; n = 34, 20-minute dwell time and two hours of negative pressure) for NPWTi utilizing Prontosan® Wound Irrigation Solution (B. Braun Medical Inc., Bethlehem, PA) were compared with data from a historical cohort of patients treated with standard NPWT (n = 74). The results showed a significant decrease in the number of debridement operations (6- and 20-minute dwell groups, P = 0·043 and P = 0·003), length of hospital stay (20-minute dwell group, P = 0·034) and time to final surgical procedure (6- and 20-minute dwell groups, P = 0·043 and P = 0·0019, respectively). An increase in the proportion of closed/covered wounds prior to discharge was significantly different between the NPWT-i groups versus the standard NPWT group (94% versus 64%, respectively; P = 0·0004).

Gabriel, Kahan, Karmy-Jones [7] performed a retrospective analysis comparing clinical outcomes of wounds treated with NPWT-i versus NPWT and the estimate cost-difference between treatments based on clinical outcomes. Results: significant differences (P < 0.0001) between NPWT-i and NPWT patients, respectively, for mean operative debridements (2.0 vs 4.4), mean hospital stay (8.1 vs 27.4 days), mean length of therapy (4.1 vs 20.9 days), and mean time to wound closure (4.1 vs 20.9 days) were found. A hypothetical economic model showed potential average reduction of $8143 for operative debridements between NPWT-i ($6786) and NPWT ($14,929) patients, a $1418 difference in average therapy costs between groups ($799/NPWT-i vs $2217/NPWT).

Fernandez, Griffiths [8] demonstrated that wound cleansing before each dressing change with a tissue-preserving fluid is an effective method to remove debris, wound exudates, and bacteria. The literature supports the use of NPWT-i as an adjunct therapy in order to decrease the number of debridement operations and their associated costs and to accelerate the wound healing process in complex surgical wounds to reduce overall length of stay.

## Case presentation

### Methods

**Figure 1:**
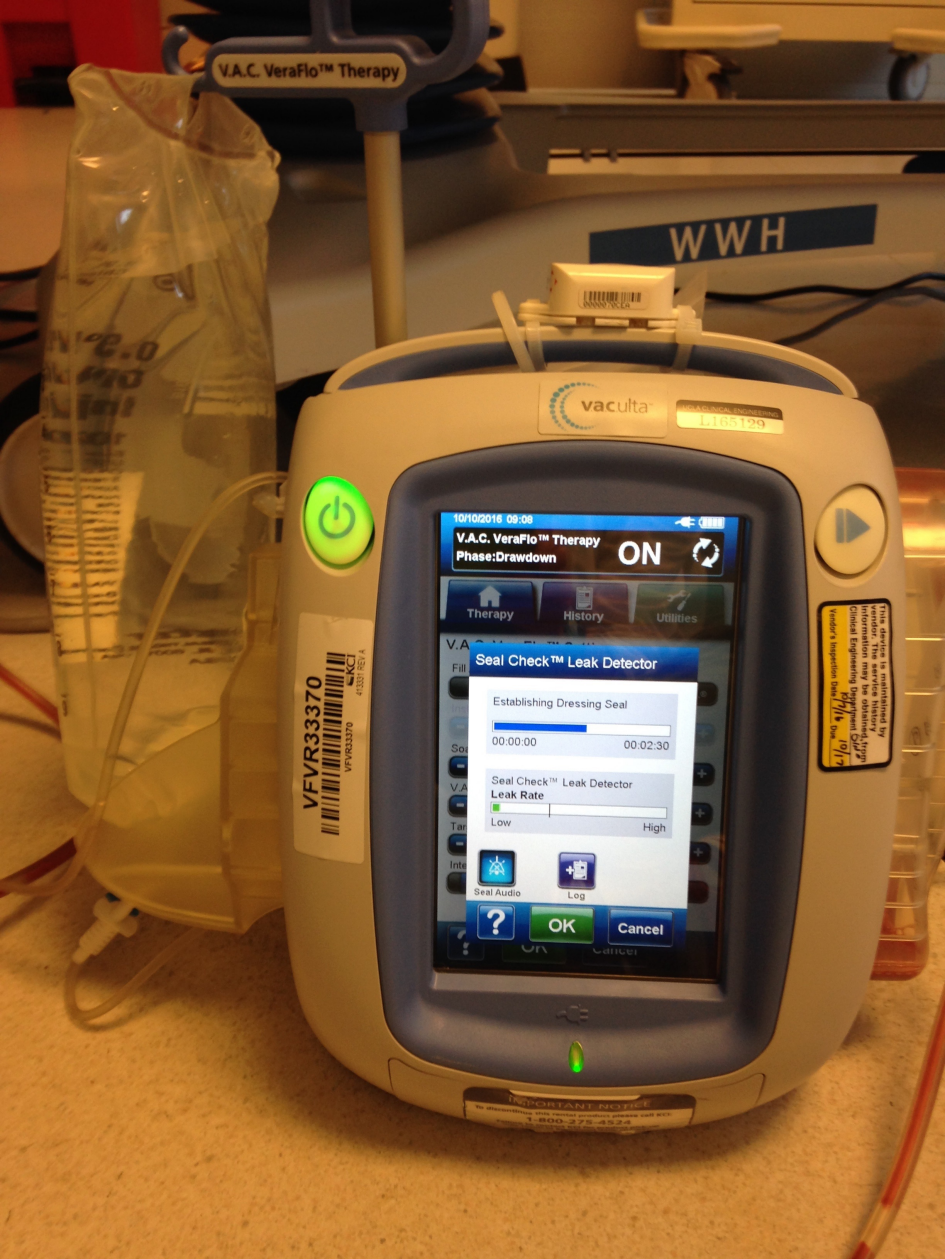
V.A.C. ULTA™ with VERAFLO™

NPWT-i (V.A.C. ULTA™ with VERAFLO™, KCI, an ACELITY Company, San Antonio, TX) (Figure 1) with either hypochlorous acid solution (HOCL) or amphotericin solution was used at approximately four cycles per day. The dwell times were five or ten minutes for the hypochlorous acid and three to four minutes for using amphotericin anti-infective solution on the wound bed. Conventional NPWT/ROCF by V.A.C.® Therapy (KCI, an ACELITY Company, San Antonio, TX) was then initiated after seven to ten days of continuous NPWT-i in four out of five patients. These retrospective case reports were done in accordance with the Declaration of Helsinki and all patients provided written informed consent for photography.

### Results

Instillation therapy elicited a faster rate of wound filling with granulation tissue and no recurrent wound infections in these complex surgical patients. 

Case Study 1

**Figure 2:**
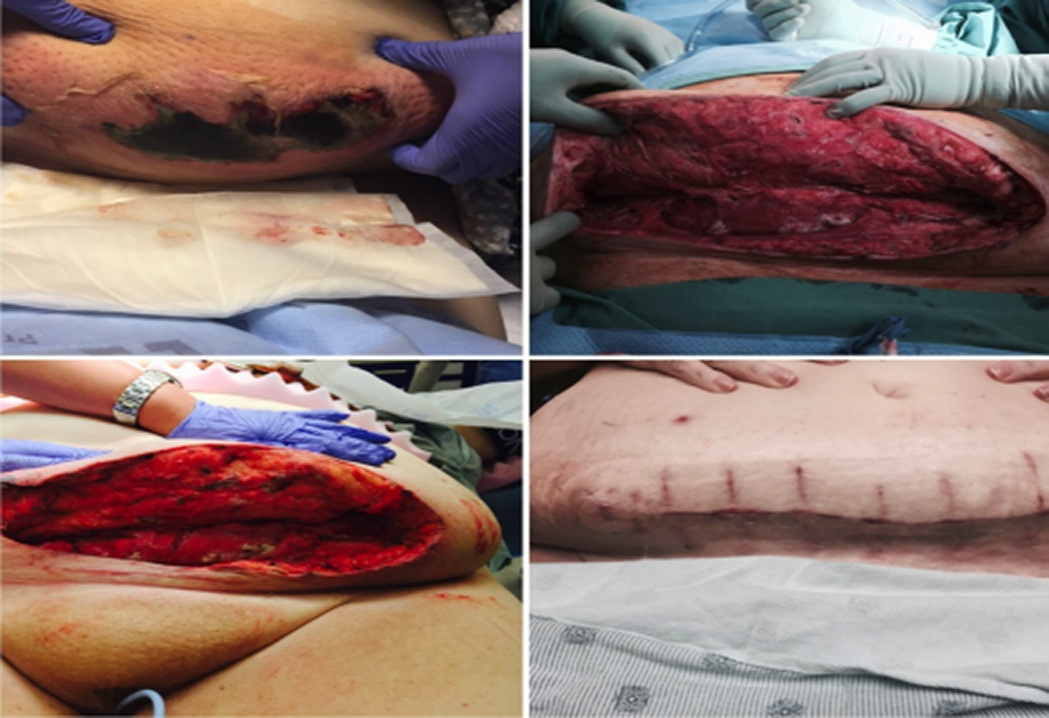
Necrotizing soft tissue infection

A 60-year-old woman POD 37 from robotic total hysterectomy and bilateral salpingo-oophorectomy for stage II endometrial adenocarcinoma developed a necrotizing soft tissue infection requiring excision and debridement resulting in a 36 cm x 10 cm x 6 cm wound (Figure 2). NPWT-i with hypochlorous acid (HOCL) was used for seven days for continued cleansing and debridement. The patient returned to the OR for delayed primary closure after application with injectable amnion/ chorion treatment in the open wound. The incisional NPWT system was applied over the 41 cm incision to promote wound closure for three weeks with weekly dressing changes. The wound healed with no further complications and the patient was able to proceed with chemotherapy six weeks after surgery.

Case Study 2

**Figure 3:**
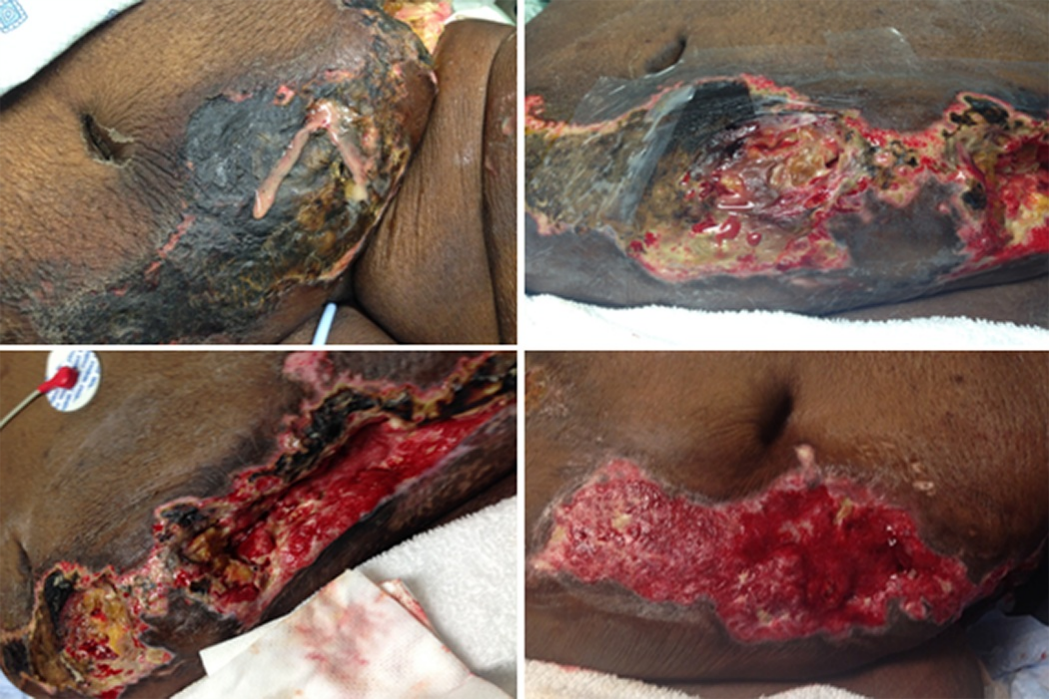
Sepsis secondary to multiple large abdominal wounds

A 35-year-old woman presented to the emergency room with sepsis secondary to multiple large abdominal wounds draining purulent, foul-smelling fluid. Her medical history included end-stage renal disease, diabetes, hypercalcemia, and chronic hepatic failure. A wound biopsy indicated calciphylaxis and a gram-positive wound infection (Figure 3). NPWT-i with HOCL was applied with a three minutes installation followed by 720 minutes negative pressure. Within 48 hours granulation tissue began to appear in the wound bed, and the patient reported less discomfort. After 54 days in the hospital the wound size had decreased 75% and the patient was discharged home with home health wound care consisting of topical dressing changes daily.

Case Study 3

**Figure 4:**
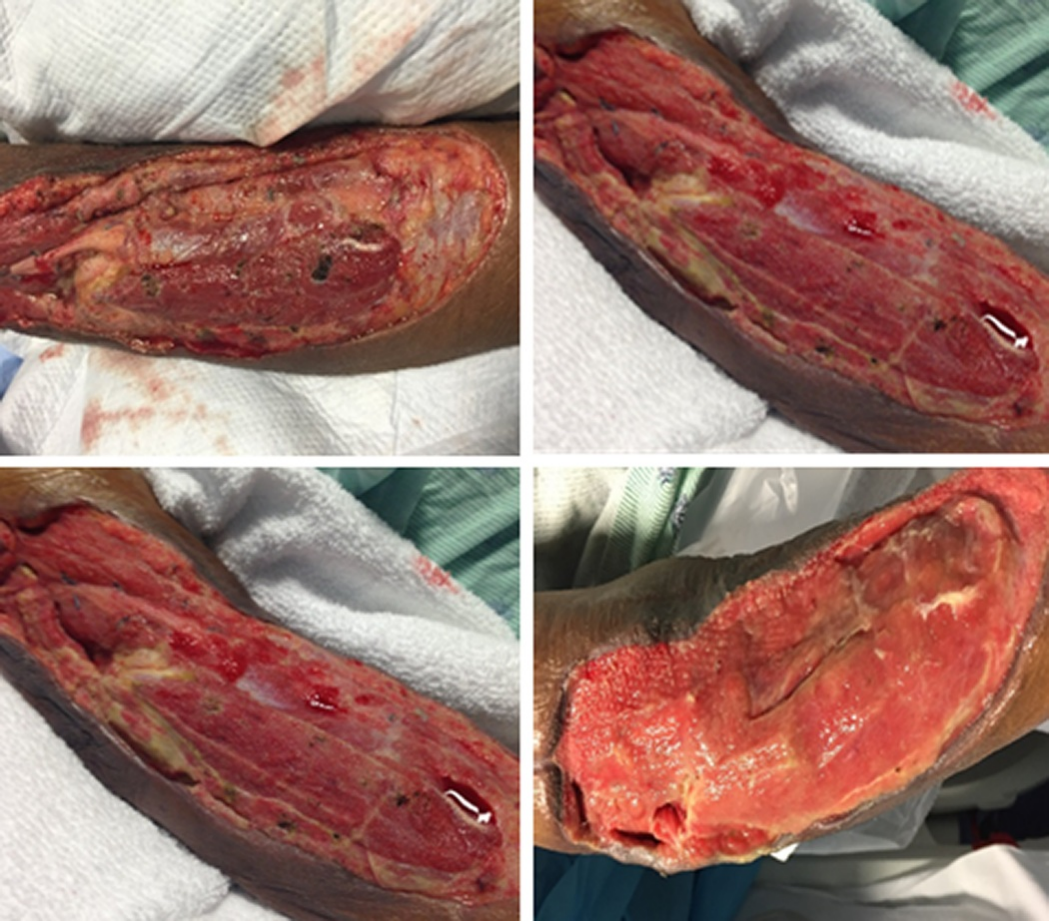
Fungal infection of right upper extremity

A 56-year-old man with alcohol and drug-induced cirrhosis received a liver transplant in July 2015 complicated by a Rhizopus species fungal infection of his right upper extremity. The patient underwent two surgical debridements including resection of the brachioradialis prior to the NPWT-i application (Figure 4). Amphotericin B was instilled in three daily cycles, three-to-four-minute dwell time for five days followed by HOCL instillation for seven days. After 27 days of combined NPWT-i and NPWT, wound closure was obtained with the assistance of a skin autograft from his right thigh.

Case Study 4

**Figure 5:**
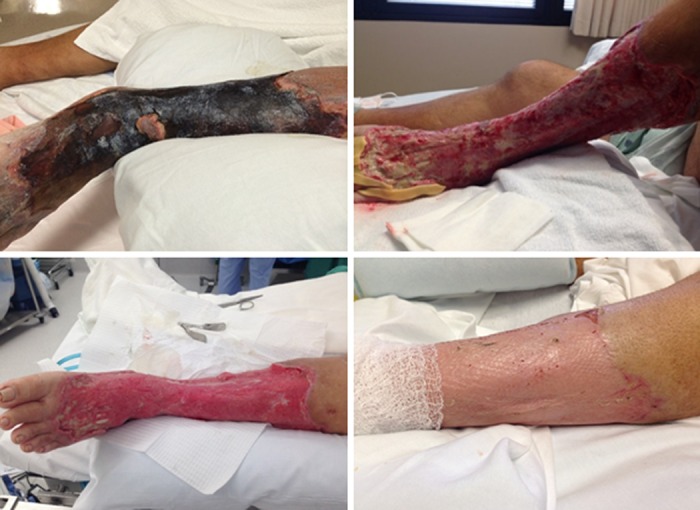
Necrotic eschar covering the left lower extremity

A 65-year-old man presented with a necrotic eschar covering the left lower extremity, sepsis, and hyperglycemia. He had been hospitalized in the Virgin Islands for the preceding two weeks and treated with antibiotics and compression wraps (Figure 5). His past medical history included uncontrolled diabetes, ethyl alcohol (ETOH) abuse, and rheumatoid disease. The patient underwent surgical debridement of necrotic tissue and the culture revealed Pseudomonas and multidrug resistant Acinetobacter. NPWT-i using HOCL with a three-to-five-minute dwell time every eight hours for 15 days was applied to the wound, and he subsequently underwent a split thickness skin graft.

Case Study 5

**Figure 6:**
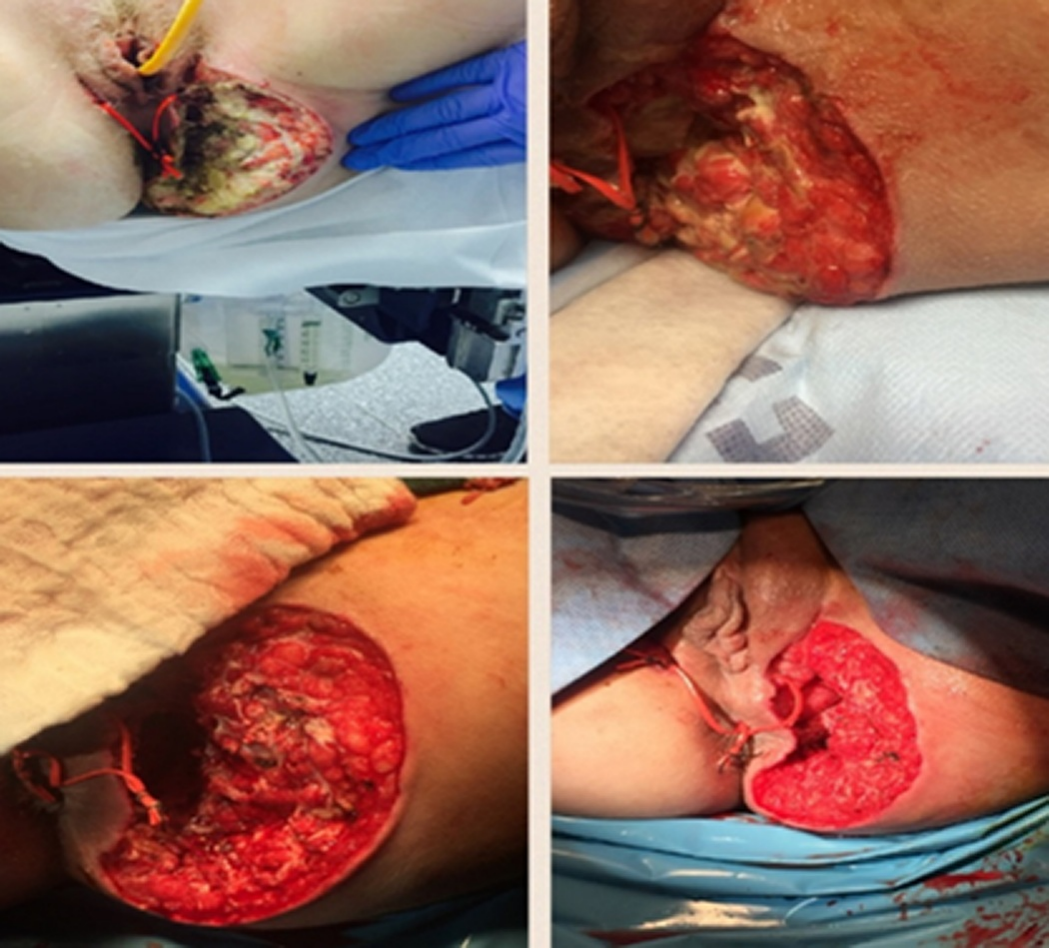
Perirectal abscess

A 23-year-old woman with antiphospholipid syndrome, systemic lupus erythematosus presented with a perirectal abscess. She was taken to the operating room (OR) for examination under anesthesia (EUA) and a seton placement after a fistulous connection was discovered from just inferior to bilateral labia majora to a left gluteal abscess (Figure 6). Multiple surgical debridements were performed subsequently after she developed necrotizing cellulitis, and the patient required a laparoscopic loop colostomy to divert the fecal stream from the wound. NPWT-i with HOCL was initiated with a six-minute dwell time and four cycles per day for eight days before she returned to the OR for EUA and amniotic chorionic membrane application to augment wound healing. After seven additional days of regular NPWT the wound was determined to be appropriate for a reconstructive left gracilis myofascial flap closure. On hospital day 29 she was ambulating, tolerating regular diet, with normal micturition and ostomy production.

## Discussion

Wound infections continue to be among the most expensive complications following surgery and are a major source of bacteria that drive the nosocomial infection rates in hospitals [9]. Wounds plagued with acute infection and biofilm are challenging problems that require an intensive multimodal approach including systemic antibiotics, appropriate surgical intervention, and advanced wound management. Since its introduction, NPWT has drastically changed the management of such surgical wounds promoting continued wound drainage and debridement while facilitating earlier granulation tissue growth. As the next iteration of this therapy, NPWT-i has proven superior in controlling the bioburden of complex infected surgical wounds while initiating these beneficial cellular effects.

In our experience, this approach was successful in obtaining source control and in decreasing healing time of complex wounds without recurrent infections. NPWT-i provides a physiologic environment that promotes healing and prepares the wound for delayed primary and secondary closure or skin grafting [5]. There is an increasing body of evidence supporting the potential clinical and financial efficacy of NPWT-i [7], and it should be strongly considered for more widespread implementation in the care of complex surgical wounds.

## Conclusions

The use of NPWT-i is an important adjunctive therapy in the management of infected complex wounds. It has been proven that granulation tissue ingrowth is essential to obtain successful soft tissue coverage [10]. NPWT-i stimulates granulation tissue, removes infectious debris, and manages bacterial bioburden.

The case studies presented herein demonstrate tangible evidence from a single tertiary care referral center that instillation therapy is effective in promoting granulation tissue and wound healing required to expedite definitive closure of complex surgical wounds. The adjunctive use of NPWT-i with surgical debridement and appropriate antibiotic therapy presents a promising multimodal therapeutic strategy for the management of infected complex wounds.
